# Performance of an Inclinometer for Inertia-Based Robotic Stability Monitoring System Under Cross-Axis Angular Deviations

**DOI:** 10.3390/s26216831

**Published:** 2026-10-29

**Authors:** Mateusz Fiedeń, Michał Olinski

**Affiliations:** Department of Fundamentals of Machine Design and Mechatronic Systems K61W10D07, Faculty of Mechanical Engineering, Wrocław University of Science and Technology, Łukasiewicza St. 7/9, 50-371 Wroclaw, Poland

**Keywords:** mechatronics, robot perception, autonomous systems, angle estimation, tip-over prevention, stability, inclinometer

## Abstract

Autonomous heavy machinery and mobile robots require accurate real-time angular state monitoring to ensure stability and prevent tip-over accidents. However, sensor mounting imperfections and operating on slopes can introduce cross-axis distortions in inclination readings. This research investigates the angle tracking accuracy and cross-axis resilience of the IFM JD1121 industrial single-axis dynamic inclinometer. Experimental validation is performed on a custom mechatronic test stand driven by a stepper motor executing a repeatable 30 s motion profile from −30° to +90°. Cross-axis interference is evaluated across automated multi-trial runs under fixed lateral angular offsets of 0°, 10°, 20°, and 30°. A preliminary field test is conducted on a mobile demolition robot using a camera-tracked ArUco marker as an optical reference. Inclinometer performance is evaluated using statistical metrics, including maximum error, mean error, mean absolute error, and SD. Quantitative cross-axis sensitivity analysis revealed low coupling coefficient, while equivalence testing (TOST) confirmed that lateral roll offsets up to 30° cause very small degradation in pitch accuracy. The preliminary field test indicated stable on-platform tracking, supporting further development of the sensor for anti-tip safety monitoring in industrial mobile platforms/robots and heavy machinery.

## 1. Introduction

The development and operation of heavy machinery in agriculture, civil construction, and mining is undergoing a steady transition from manual human piloting toward teleoperation and autonomous execution [1,2]. Modern mobile robotic platforms are required to independently perceive their internal kinematic state to ensure operational safety during complex operations [2,3,4]. When heavy equipment operates without direct human physical feedback, identifying critical vehicle parameters and body orientation becomes a core requirement for microprocessor-based stability systems [5].

**Figure 1:**
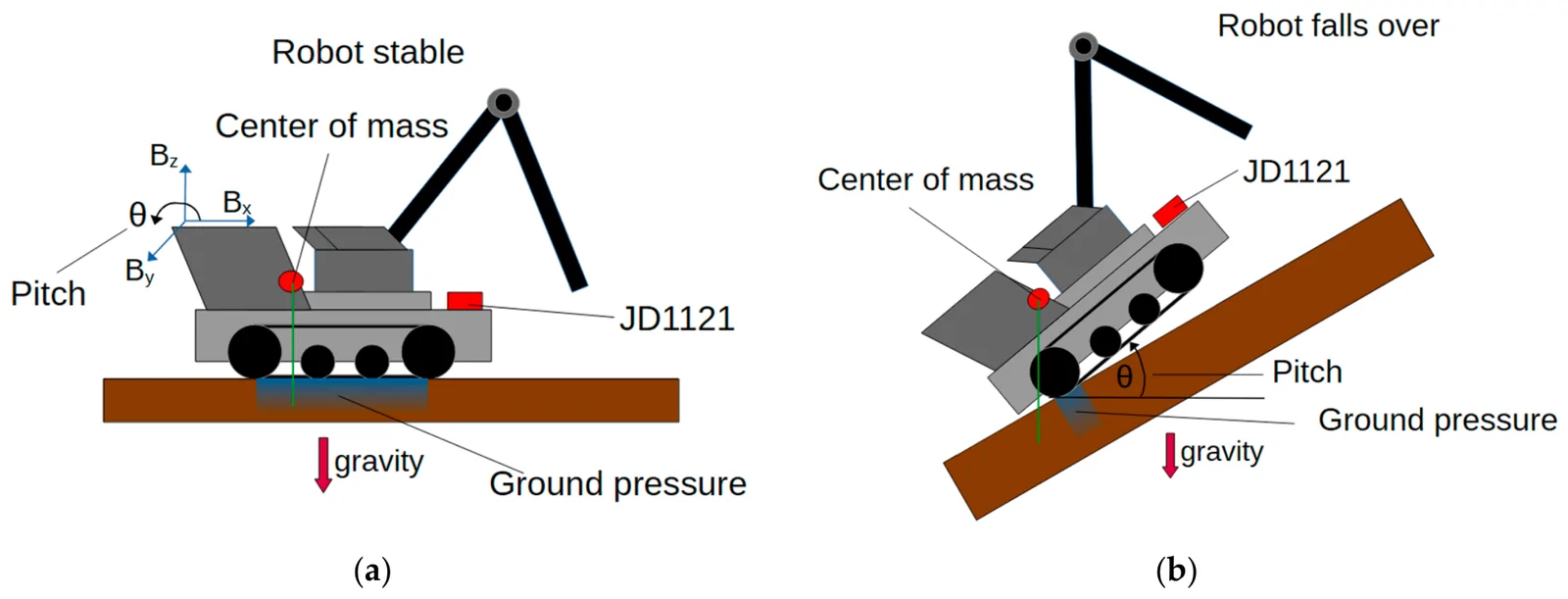
Scheme explaining the concept of stability for a mobile robot with marked center of mass and ground support area: (**a**) Robot stable. (**b**) Robot losing stability.

Maintaining tip-over stability on uneven terrain represents a fundamental safety challenge for mobile robots and heavy machinery. In particular, severe vehicle longitudinal pitching caused by irregular ground profiles or high-load manipulator movement is the main cause of tipping accidents [6]. To evaluate the likelihood of rollover or pitching, several mathematical criteria and stability metrics are commonly employed. The classic geometric approach relies on evaluating the projection of the center of mass onto the support base boundary (Figure 1a,b) to assess static and quasi-static tipping limits [7,8,9]. To account for dynamic forces during motion, the Force-Angle measure incorporates both gravitational and dynamic acceleration vectors acting on the machine [10]. Furthermore, dynamic overturn prevention algorithms use real-time kinematic models to predict instability and compute preventive control actions before tipping occurs [11]. In control theory literature, the modeling, stabilization, and observer-based feedback control of nonlinear dynamic systems and mobile robots have been widely investigated to ensure robust performance under external disturbances [12,13]. These control strategies often rely on advanced state estimation algorithms and observer feedback to reconstruct unmeasured system states and compensate for dynamic uncertainties.

Recent studies have further investigated the dynamic behavior of mobile platforms on uneven terrain, highlighting how terrain-induced changes in vehicle attitude affect wheel–ground interactions, dynamic stability margins and overturning thresholds [14,15]. In mobile manipulators, severe dynamic coupling between onboard arms and mobile bases requires real-time tip-over avoidance algorithms, such as extended Zero-Moment Point (ZMP) approaches and improved tip-over moment criteria [16,17]. Furthermore, stability-oriented control strategies and terrain-adaptive mobile platforms operating in unstructured or inclined environments [18,19] underscore the importance of reliable attitude estimation for maintaining overturning stability.

In all cases, for effective safety control, onboard sensors must deliver high-precision real-time measurements of pitch angle relative to the gravity vector [20,21]. A broad range of sensing technologies is used to track kinematic states in mobile machines, including optical sensors, odometry encoders, and inertial measurement units (IMUs) [22,23]. Wireless multi-axis IMUs combining accelerometers, gyroscopes, and magnetometers are frequently applied in human motion characterization, biomechanics, and mobile robotics due to their small physical dimensions and high update rates [22,24]. While their inclination-related performance under dynamic conditions can be affected by multi-axis angular motion [25], combining measurements from multiple MEMS accelerometers and gyroscopes has been proposed to enhance inclination sensing accuracy [26]. Moreover, in heavy machinery applications, raw inertial measurements are subject to structural vibrations and dynamic accelerations, requiring specialized filtering, redundancy, and calibration [27,28]. Additionally, while multi-axis IMU hardware measures raw angular rates and linear accelerations, orientation estimation algorithms relying on Euler angle representations suffer from mathematical singularities (gimbal lock) near vertical orientation angles. Furthermore, three-axis orientation sensors frequently rely on magnetometers, for heading reference. Under real-world operating conditions, these sensors can be affected by magnetic fields or metal objects in the surrounding environment, potentially coupling distortion into the estimated inclination angles.

Industrial dynamic inclinometers offer narrow noise bands and high signal reliability. However, mobile machines in field environments rarely operate on perfectly level ground-vehicles frequently execute tasks while positioned on uneven terrain [3,29,30]. When such sensors measure pitch inclination under a non-zero lateral tilt, cross-axis sensitivity, defined as the coupling between orthogonal sensing axes, can introduce systematic measurement errors and zero-point offsets [31], potentially degrading the accuracy. Dynamic tilt testing methodologies, such as those based on conical motions, have demonstrated that multi-axis angular vibrations can influence the frequency response and drift of MEMS inclinometers under non-level conditions [25]. In parallel, physical modeling and reliability analysis frameworks incorporating robot motion smoothness and dataset variability [32] may inform the validation strategy for safety-critical monitoring architectures.

Our initial tests comparing IMUs and inclinometers [33] established baseline sensor behavior under ideal horizontal orientation (0° roll), but did not address possible systematic measurement degradation caused by spatial cross-axis coupling. Although previous studies have examined inclination sensing and MEMS sensor testing, the quantitative characterization of cross-axis sensitivity in industrial dynamic inclinometers operating under sustained non-zero transverse slopes remains limited. In particular, it remains unclear whether lateral roll offsets introduce persistent pitch-angle bias, reduce linearity, or alter the dynamic error response of embedded filtering algorithms. This paper presents an extended evaluation of an industrial dynamic inclinometer (JD1121, ifm electronic gmbh, Essen, Germany) on a mechatronic test stand with an optically validated angular reference. The primary objective of this study is to quantify the pitch angle tracking accuracy and signal stability under three initial lateral tilt deviations (10°, 20°, and 30°), thereby evaluating cross-axis sensitivity effects and establishing operational reliability boundaries for stability monitoring systems in mobile robots. To evaluate experimental repeatability and measurement consistency, experimental data are collected across 10 test runs per setup and subjected to statistical analysis, evaluating metrics such as maximum error and mean absolute error. Furthermore, this work presents preliminary field evaluation and initial experimental results for the sensor unit mounted on a mobile demolition robot. Overall, these investigations represent a step toward developing an autonomous stability monitoring system for safe vehicle operation in harsh, unstructured environments.

## 2. Materials and Methods

**Figure 2:**
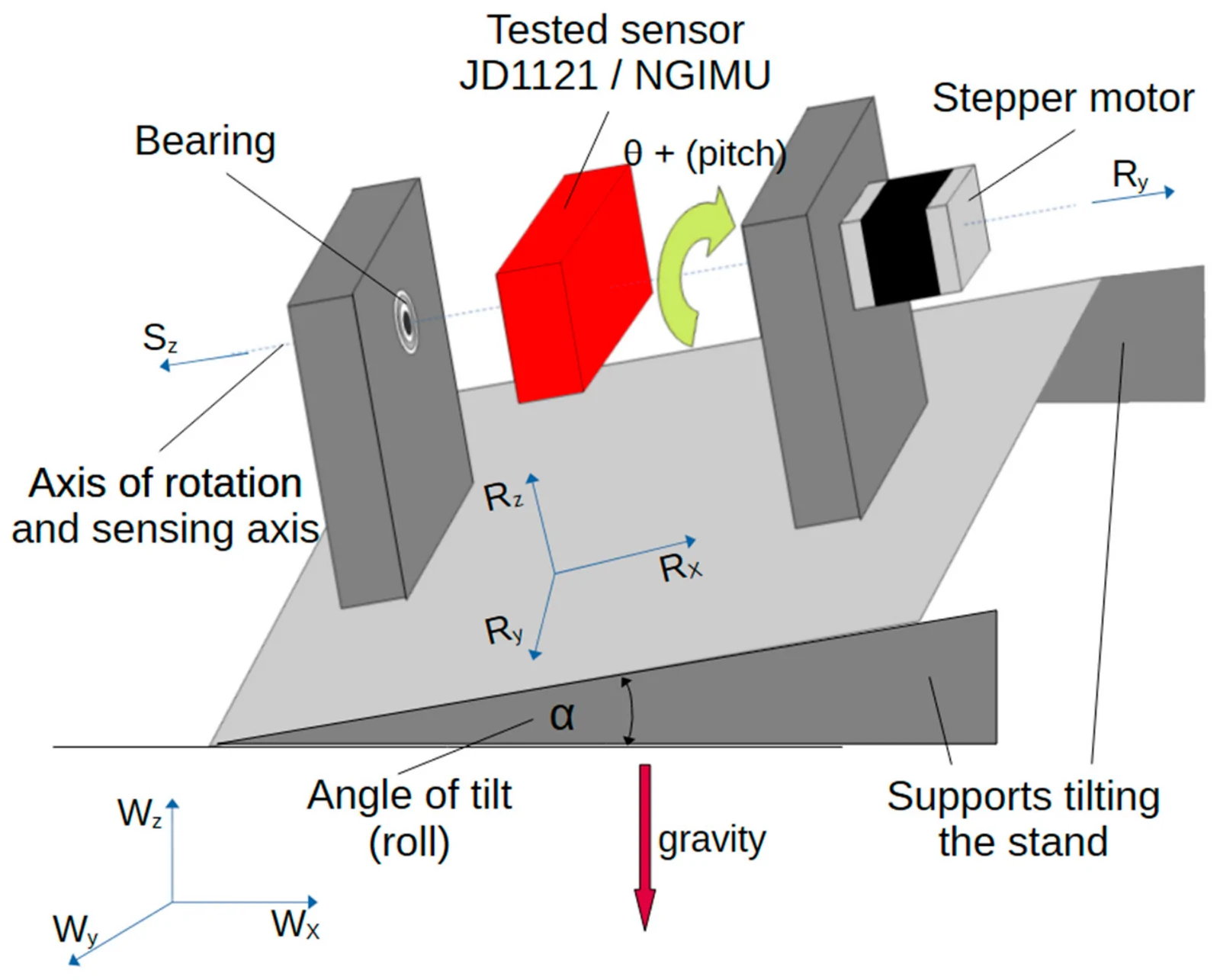
Scheme of the mechatronic test stand, indicating the primary axis of rotation, mounted sensor, stepper motor drive, bearing and supports for tilting the lateral deviation angle. Arrows indicate the gravity vector *g*, the motor axis *R_y_*, the positive directions of the platform angle *θ* (pitch) and the lateral deviation *α* (roll), and the sensing axis *S_z_* of the inclinometer.

To select suitable onboard instrumentation for mobile robots, sensor performance must be evaluated under operational conditions. In a preliminary comparative study conducted on a level ground surface, the performance of a 3-axis NGIMU (x-io Technologies Ltd., Bristol, UK) was evaluated alongside an IFM JD1121 single-axis industrial inclinometer (Figure 2). Initial findings revealed that the NGIMU provided adequate dynamic tracking within moderate pitch ranges (0° to 80°), obtaining a maximum error of 1.138° and a mean absolute error of 0.315°. However, its internal orientation estimation algorithm degraded near vertical inclinations. Above 80° pitch, mathematical singularities associated with gimbal lock caused coordinate sequence flips, driving the maximum measurement error up to 5.084°. In contrast, the JD1121 industrial inclinometer demonstrated high signal stability and continuous accuracy across the entire 0° to 90° motion profile, maintaining a maximum error of 1.060° and a mean absolute error of 0.335° [33]. Furthermore, the NGIMU makes active use of a magnetometer, which, under real-world operating conditions, may introduce random errors that are heavily dependent on the specific operating environment. Consequently, the industrial inclinometer was selected for detailed evaluation under cross-axis angular deviations, forming the focus of this work.

**Figure 3:**
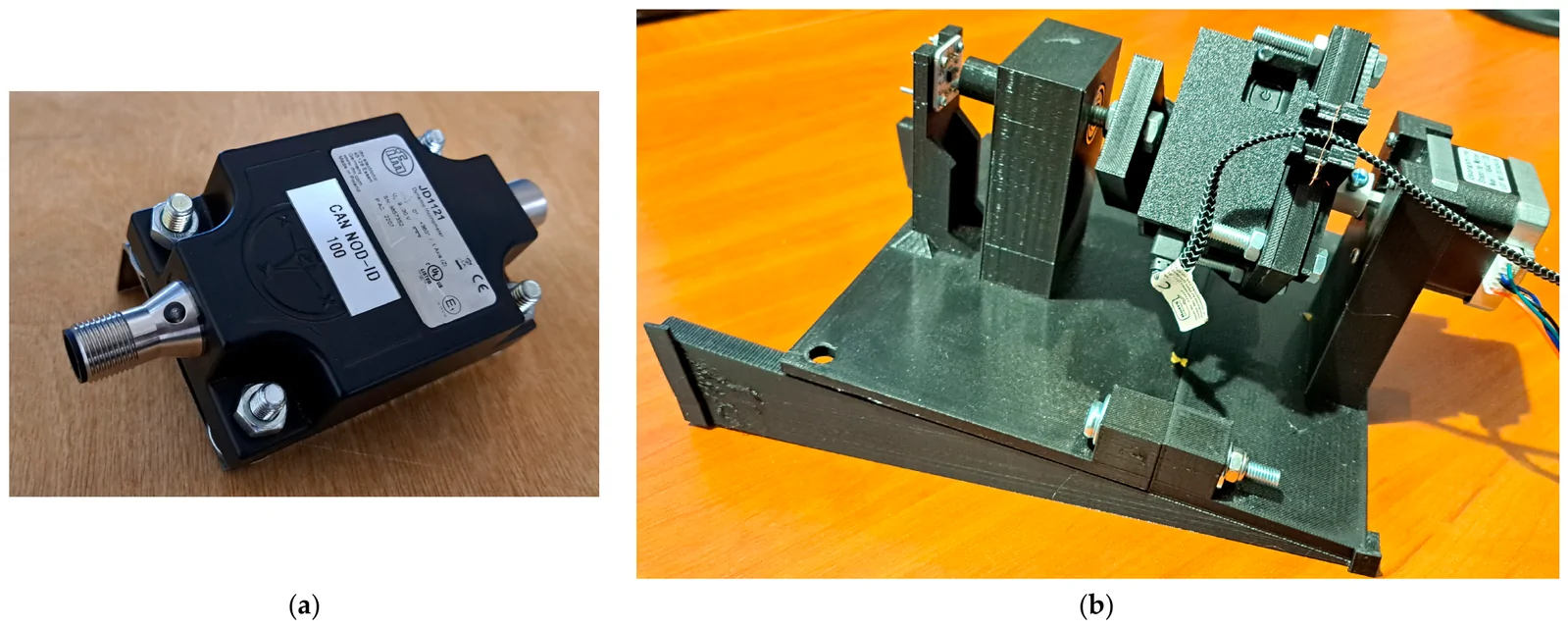
Physical workstation: (**a**) JD1121 industrial inclinometer unit. (**b**) Assembled stationary mechatronic test stand with structural frame, mounted sensor, and stepper motor drive assembly.

The physical sensor unit evaluated in this work is therefore the IFM JD1121 dynamic inclinometer (comprising a single-axis accelerometer and gyroscope), shown in Figure 3a. Designed specifically for heavy-duty machinery applications, the sensor operates across a continuous single-axis measurement range from 0° to 360° with a factory static accuracy rating of ±0.3°. Physical internal damping is integrated into the sensor architecture to reduce high-frequency mechanical vibrations without relying on complex external filter tuning. Sensor readings are transmitted via CAN bus interface with a parameterizable angular resolution set to 0.01°. The internal MEMS accelerometer unit operates within a dynamic measurement range of ±4 g, providing robust tilt sensing capabilities under dynamic operational loads.

To evaluate the cross-axis sensitivity and pitch estimation stability of the sensor under initial lateral tilt conditions, experiments were executed on a custom-designed mechatronic test rig shown schematically in Figure 2. The physical experimental setup, illustrated in Figure 3b, consists of a structural frame and rotating platform additively manufactured from PET-G. Actuation is provided by a 17HS4401 bipolar stepper motor (1/32 microstepping, 0.05625°/step); its commanded position served as the angular reference, whose accuracy was verified by an independent optical measurement (see below and Table 1). To introduce systematic cross-axis lateral deviations, the test stand assembly was mounted on triangular structural supports, establishing fixed initial lateral roll offsets of 10°, 20°, and 30° relative to the horizontal ground plane. Both data streams were timestamped by a single system clock; the inclinometer data (~110 Hz at rest, ~190 Hz during motion) were paired with the first subsequent reference sample (mean offset ≈ 3 ms). No latency compensation was applied; the reported errors therefore include the intrinsic latency of the sensor.

Experimental testing was performed under controlled laboratory conditions at room temperature. For each of the four initial lateral tilt configurations (0°, 10°, 20°, and 30°), the measurement procedure was repeated across 10 identical automated trials to ensure experimental repeatability and measurement consistency. Before each trial, the mean inclinometer reading over ~2 s at rest was subtracted from all readings of that trial; as the initial platform position was not referenced to a fixed datum, the results characterise the tracking of angle changes rather than absolute inclination. The test platform was rotated at a constant angular velocity of approximately 9°/s over a planned movement cycle lasting around 30 s. The rotation profile comprised five distinct operational phases: an initial positive rotation from 0° to +90° over 10 s; a stationary hold period at +90° for 1.3 s to assess steady-state signal settling; a reverse rotation from +90° down to −30° over 13.3 s; a secondary hold period for 1.3 s; and a final return rotation from −30° back to the origin at 0° over 3.3 s.

The world frame *W* is defined with the *z*-axis pointing upwards, opposite to the gravity vector *g*. The test-rig frame *R* is obtained by rotating *W* about its *x*-axis by the lateral deviation angle *α* (roll; *α* = 0°, +10°, +20°, +30°), imposed statically by the supports; the positive direction of *α* is indicated in Figure 2. The platform frame *P* is obtained by rotating *R* about its *y*-axis, which coincides with the motor axis, by the platform angle *θ* (pitch); positive *θ* corresponds to the positive direction of the commanded motor rotation, i.e., towards the +90° position indicated in Figure 2. The rotation sequence is therefore roll followed by pitch, *R*_WP_ = *R_x_*(*α*)·*R_y_*(*θ*). The inclinometer is a single-axis sensor measuring the rotation about its *z*-axis, perpendicular to its mounting surface, over the range of 0–360° with a resolution of 0.01°; it was mounted vertically on the platform with its *z*-axis parallel to the motor axis. As the inclinometer output decreases for positive *θ*, its sign was inverted to match the reference convention. Both *θ* and the inclinometer output were expressed relative to the initial position of each trial.

**Figure 4:**
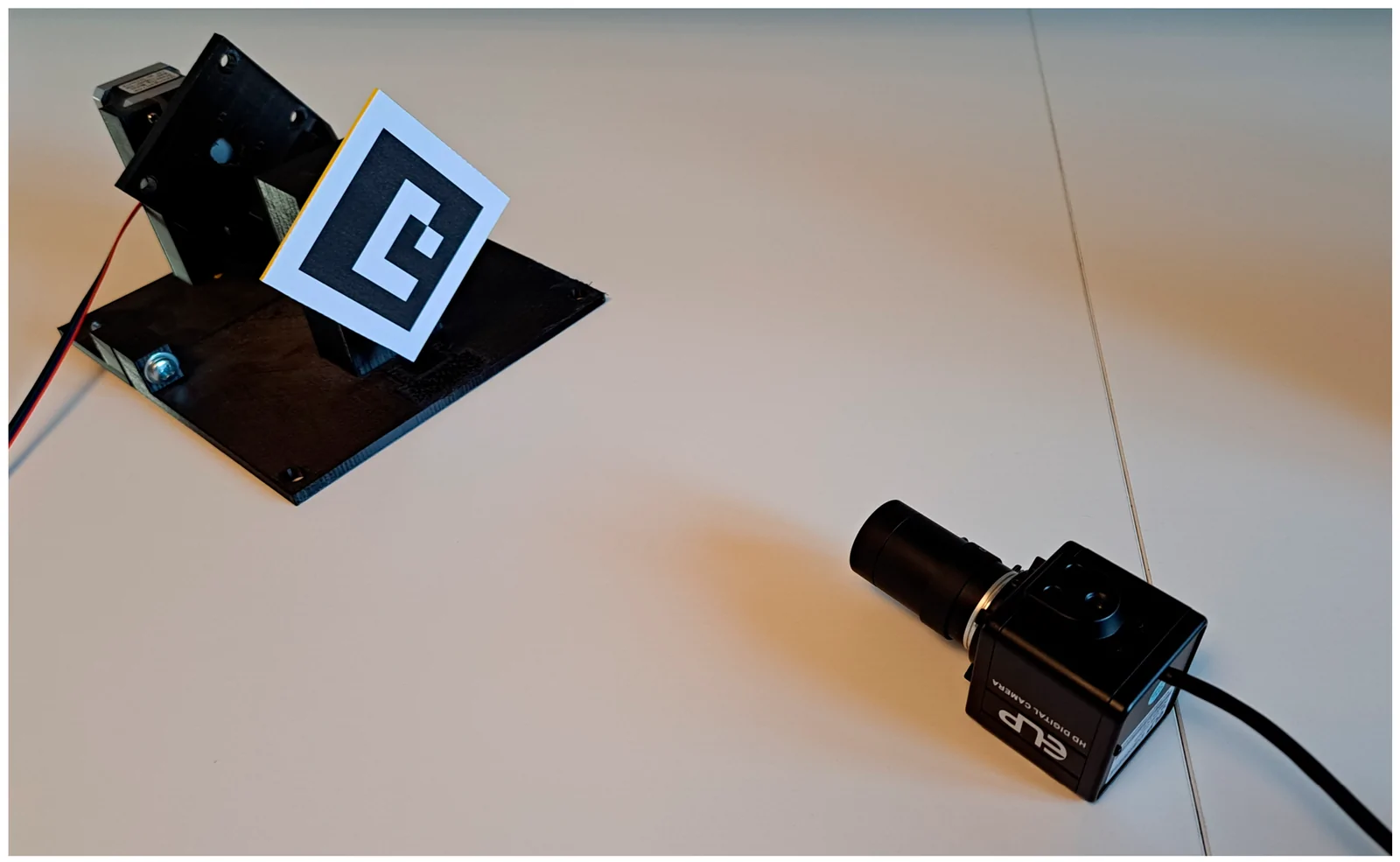
Validation of the measurement station using a camera and the ArUco marker.

Since the angular position of the platform was derived from the commanded stepper-motor position, the reference was validated using an independent optical measurement. An ArUco marker (DICT_4X4_50, side length 63.75 mm) was rigidly attached to the rotating platform and tracked by a 4K USB camera with a 5–50 mm varifocal lens (Sony IMX317 sensor; ELP, Shenzhen Ailipu Technology Co., Ltd., Shenzhen, China) (800 × 600 px, MJPG, ~30 fps, fixed exposure of 10 ms, autofocus disabled), calibrated with a ChArUco target (9 × 6 squares, reprojection RMS error 0.21 px), shown in Figure 4.

The commanded angle was logged by the controller at ~178 Hz. Both angles were expressed relative to the initial position, with the zero of the optical measurement defined as the mean over the initial dwell phase and compared at corresponding time instants without latency compensation. The platform executed the motion profile used in the main experiments in 10 consecutive runs at 0° lateral deviation. Over the 10 runs, the mean dwell deviations between the optically measured and the commanded angle were 0.07 ± 0.08° at −30°, −0.09 ± 0.02° at +90° and 0.00 ± 0.01° at 0° after the full profile (mean ± SD). During motion, a direction-dependent deviation of approximately ±0.2° was observed, consistent with a constant camera acquisition latency of 22.5 ± 0.2 ms; it vanished during dwell phases and was therefore not attributed to the reference.

The uncertainty budget (Table 1) was derived from the dwell phases of the 10 runs. For each run and dwell position, the dwell deviation was computed as the mean difference between the optically measured and the commanded angle over the dwell phase (~1.3 s, ~40 camera frames). The maximum systematic deviation is the largest run-averaged dwell deviation (0.095° at +90°), treated as the half-width of a rectangular distribution. The dwell repeatability is the pooled standard deviation of the −30° and +90° dwell deviations across runs. The zero return is the RMS deviation at 0° after the full profile. The hysteresis bound was obtained from a least-squares fit including a direction-dependent term. Together with the step resolution, these give a combined standard uncertainty of *u*_ref_ = 0.08° (*U* = 0.17°, *k* = 2).

The reprojection RMS error of 0.21 px characterises the quality of the intrinsic camera calibration and does not translate directly into the angular accuracy of the reference, which additionally depends on the marker detection, its size in the image and the pose estimation. The measurement noise of the optical system was therefore not treated as a separate component: it is contained in the scatter of the dwell deviations across runs and thus in the dwell repeatability component of Table 1.

Evaluation of the inclinometer performance was conducted by comparing the measured pitch angle against the reference trajectory given by the commanded stepper-motor position across all recorded data points. The statistical indicators used to evaluate sensor performance include the instantaneous measurement error (*e_i_*), maximum absolute error (*e*_max_), mean error (ME), and mean absolute error (MAE), calculated according to the mathematical formulas presented in Equation (1):

(1) $$e_{i}=\theta_{i}-\theta_{\mathrm{ref},i};\;e_{\max}=\mathrm{MAX}|\theta_{i}-\theta_{\mathrm{ref},i}|\;\mathrm{ME}=\frac{1}{n}\sum_{i=1}^{n}\left(\theta_{i}-\theta_{\mathrm{ref},i}\right);\;\mathrm{MAE}=\frac{1}{n}\sum_{i=1}^{n}\left|\theta_{i}-\theta_{\mathrm{ref},i}\right|$$

where the instantaneous measurement error *e_i_* represents the angular difference in sensor reading at each sample point (*θ_i_*) relative to the reference motor position (*θ*_ref,*i*_), and the metrics *e*_max_, ME, and MAE evaluate the maximum deviation, systematic directional bias, and average absolute magnitude of error across the total sample count of each trial, respectively.

To quantify the cross-axis sensitivity, the coefficient S_CA_ is defined as the change in the mean pitch error per unit of lateral (roll) deviation, in Equation (2):

(2) $$S_{\mathrm{CA}}=\;\frac{∆\mathrm{ME}}{∆\alpha }$$

where α is the lateral deviation angle. S_CA_ [°/°] was estimated as the slope b_1_ of the linear regression ME*_k_* = *b*_0_ + *b*_1_•*α_k_* + *ε_k_* fitted to the per-trial mean errors of all trials (*k* = 1…40), using heteroscedasticity-consistent (HC3) standard errors.

## 3. Results

### 3.1. Baseline Measurement Results at 0° Lateral Deviation

**Figure 5:**
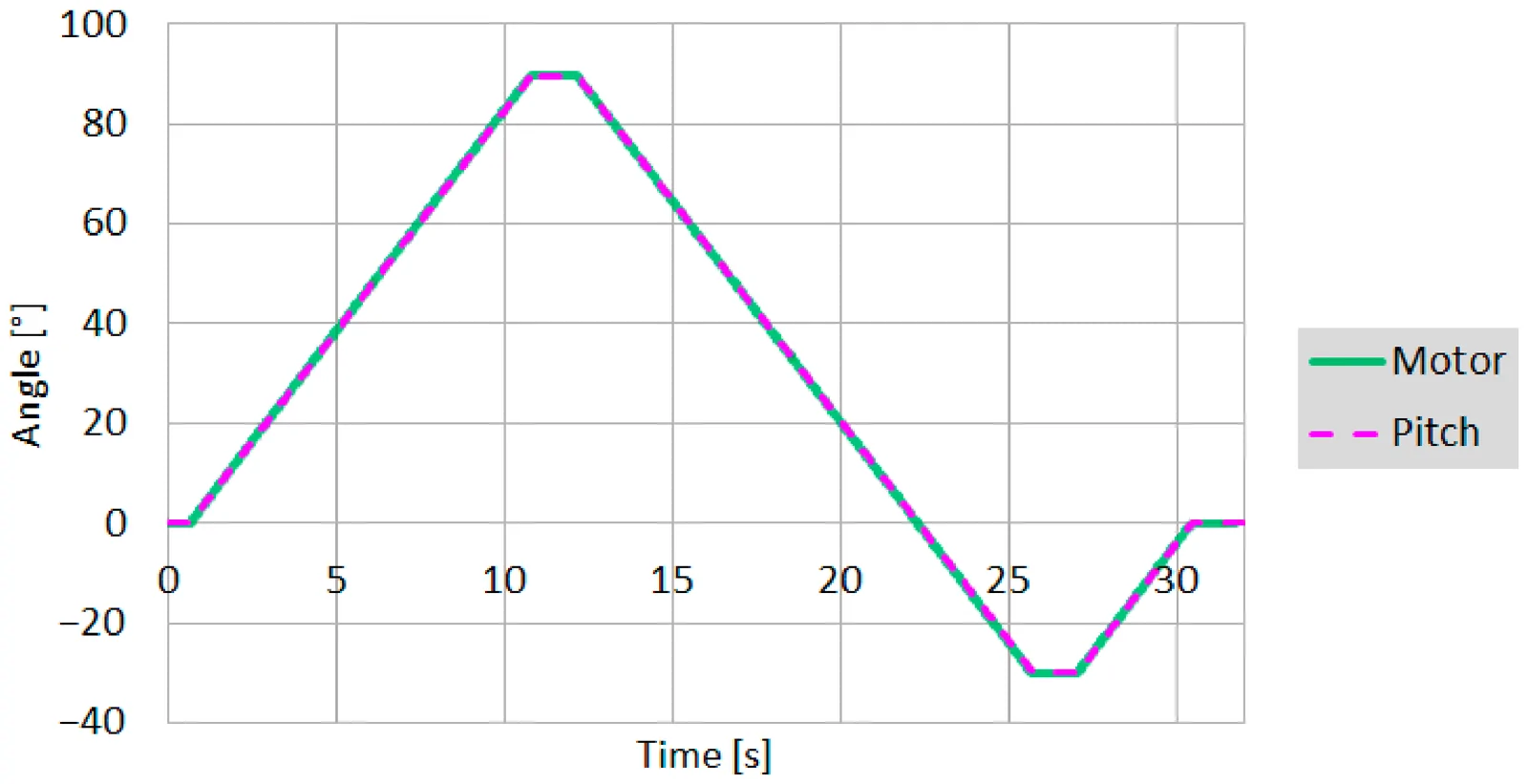
Measured angle in comparison to the motor reference trajectory at 0° lateral deviation.

**Figure 6:**
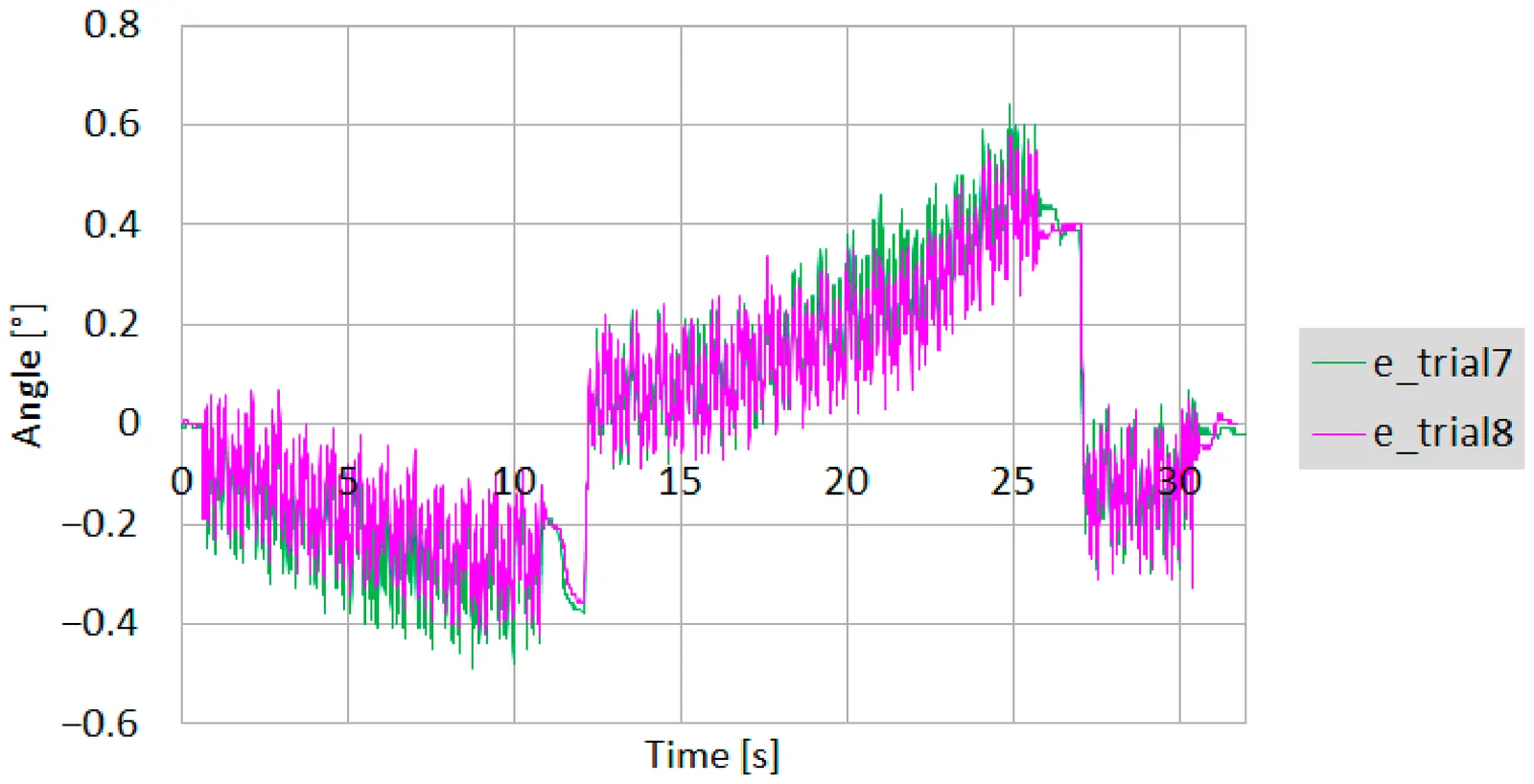
Measurement error *e_i_* profile during the 30 s movement cycle at 0° lateral deviation.

The baseline pitch tracking performance of the JD1121 inclinometer under zero lateral roll offset (0°) is illustrated in Figure 5. The recorded output closely follows the reference trajectory across all phases of the 30 s cycle. The instantaneous measurement error *e_i_* recorded throughout the baseline test is shown in Figure 6, together with additional error from another trial.

During the stationary hold periods at +90° and −30°, the measurement error settled to a low static offset (below 0.4°), which diminished as the platform moved back towards intermediate angles (near 0°). During motion, the error increased gradually within each motion segment, reaching its largest values near the extreme pitch angles (e.g., 0.640° and 0.580° near −30° in Figure 6), and changed sign abruptly at each direction reversal. The largest values among the 10 trials were *e*_max_ = 1.060°, MAE = 0.335° and ME = −0.148° (all in trial 1), while the means over all 10 trials were *e*_max_ = 0.661°, MAE = 0.214° and ME = −0.008° (Table 2).

### 3.2. Measurement Results Under 10° Cross-Axis Angular Deviation

**Figure 7:**
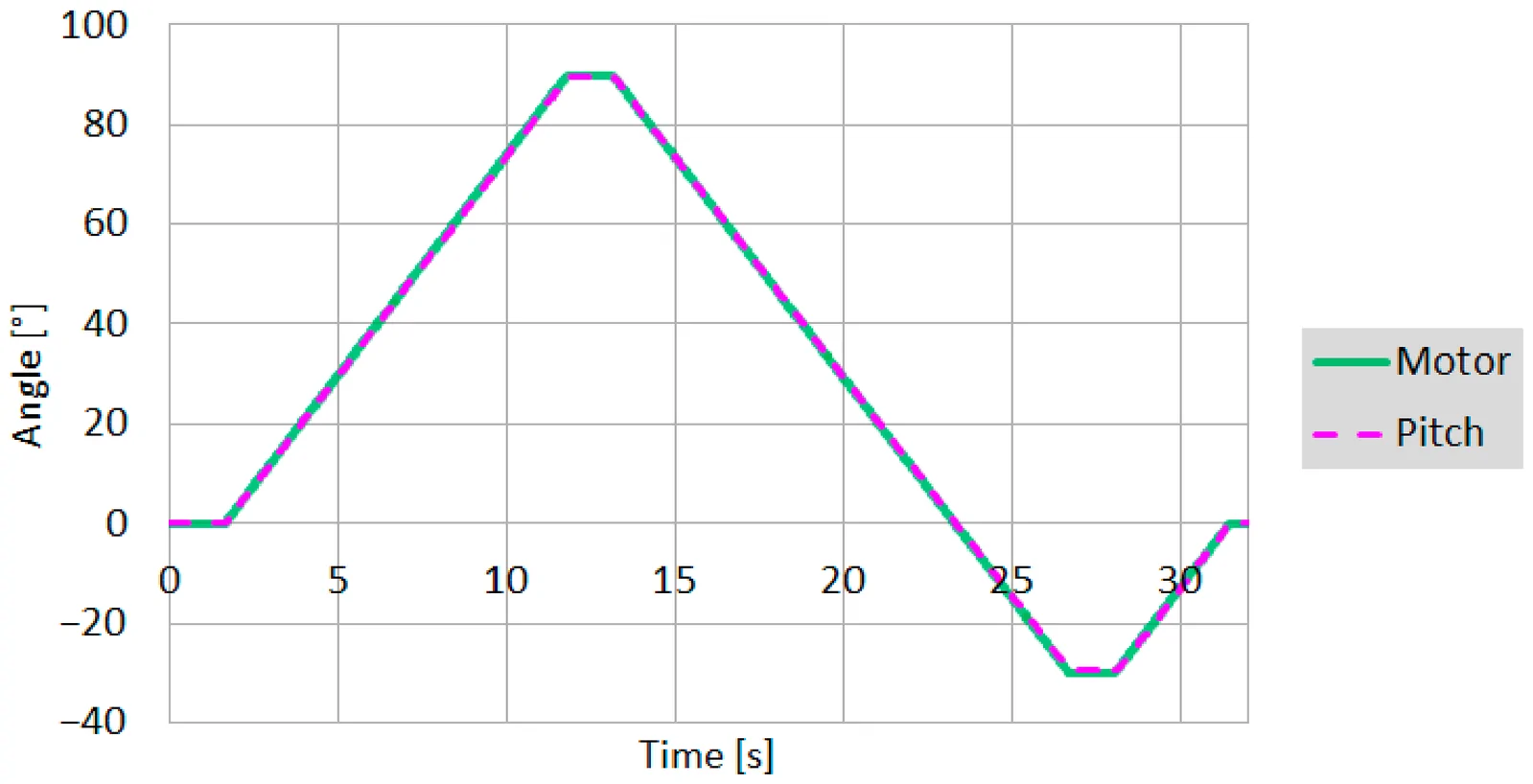
Measured angle against the reference trajectory under 10° lateral deviation.

**Figure 8:**
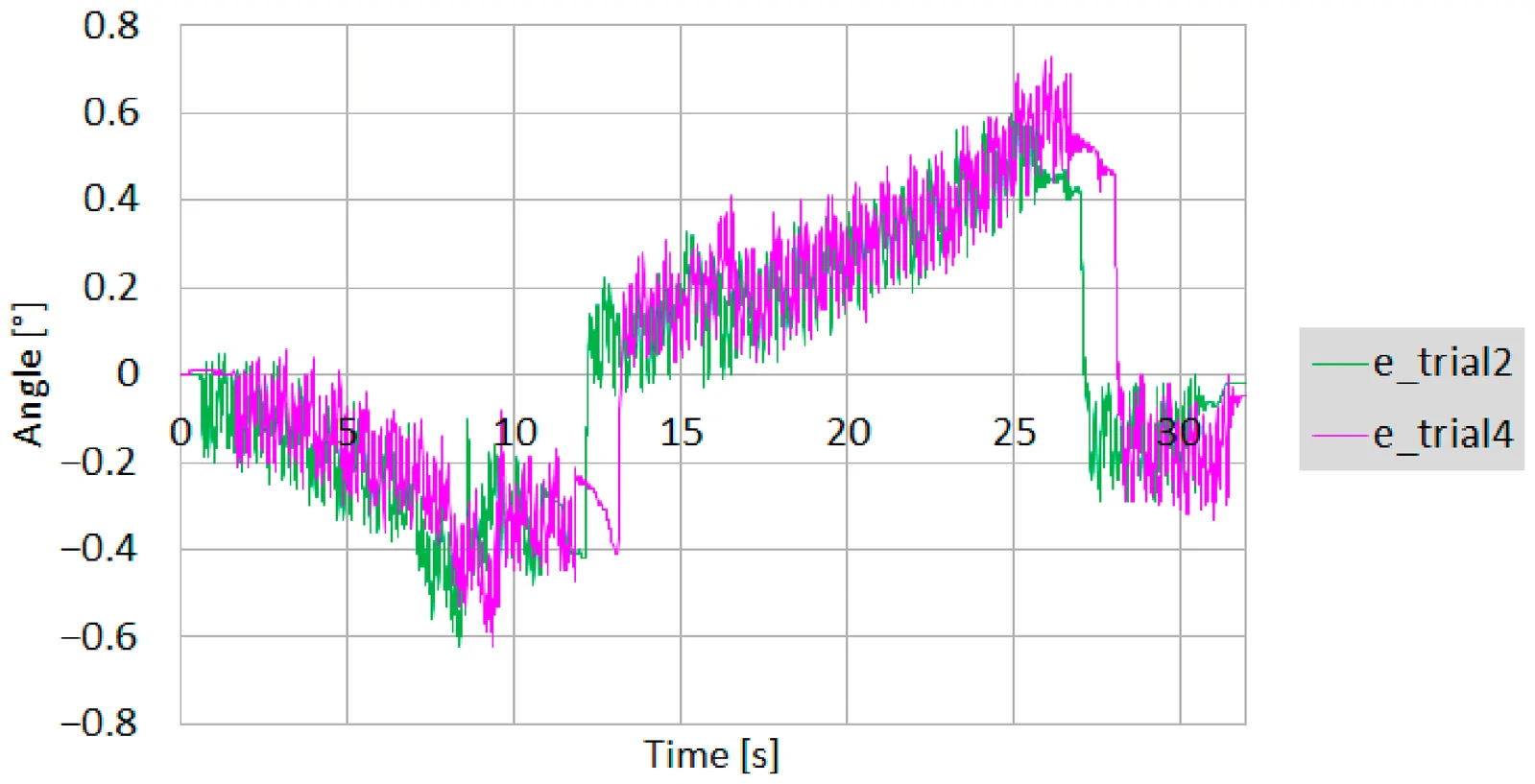
Measurement error *e_i_* profile during the 30 s movement cycle under 10° lateral deviation.

Evaluating sensor performance under a 10° initial lateral roll deviation illustrates the early effects of cross-axis orientation on pitch tracking. The time-series trajectory comparing the sensor output with the reference motor position is shown in Figure 7, with the corresponding instantaneous error *e_i_* profile presented in Figure 8 (again with error from another trial).

Similarly to the baseline condition, the inclinometer tracked the reference across the full motion cycle. Under the 10° lateral deviation, the error increased gradually during each motion segment, reaching approximately −0.5° near +90° and +0.5° near −30°, and changed sign abruptly at each direction reversal (Figure 8). The offset persisted during the stationary hold phases and diminished as the platform returned towards intermediate angles; the peak instantaneous errors remained below 1.0°. The largest values among the 10 trials were *e*_max_ = 0.760°, MAE = 0.299° and ME = −0.144°.

### 3.3. Cross-Axis Angular Deviation of 20°

**Figure 9:**
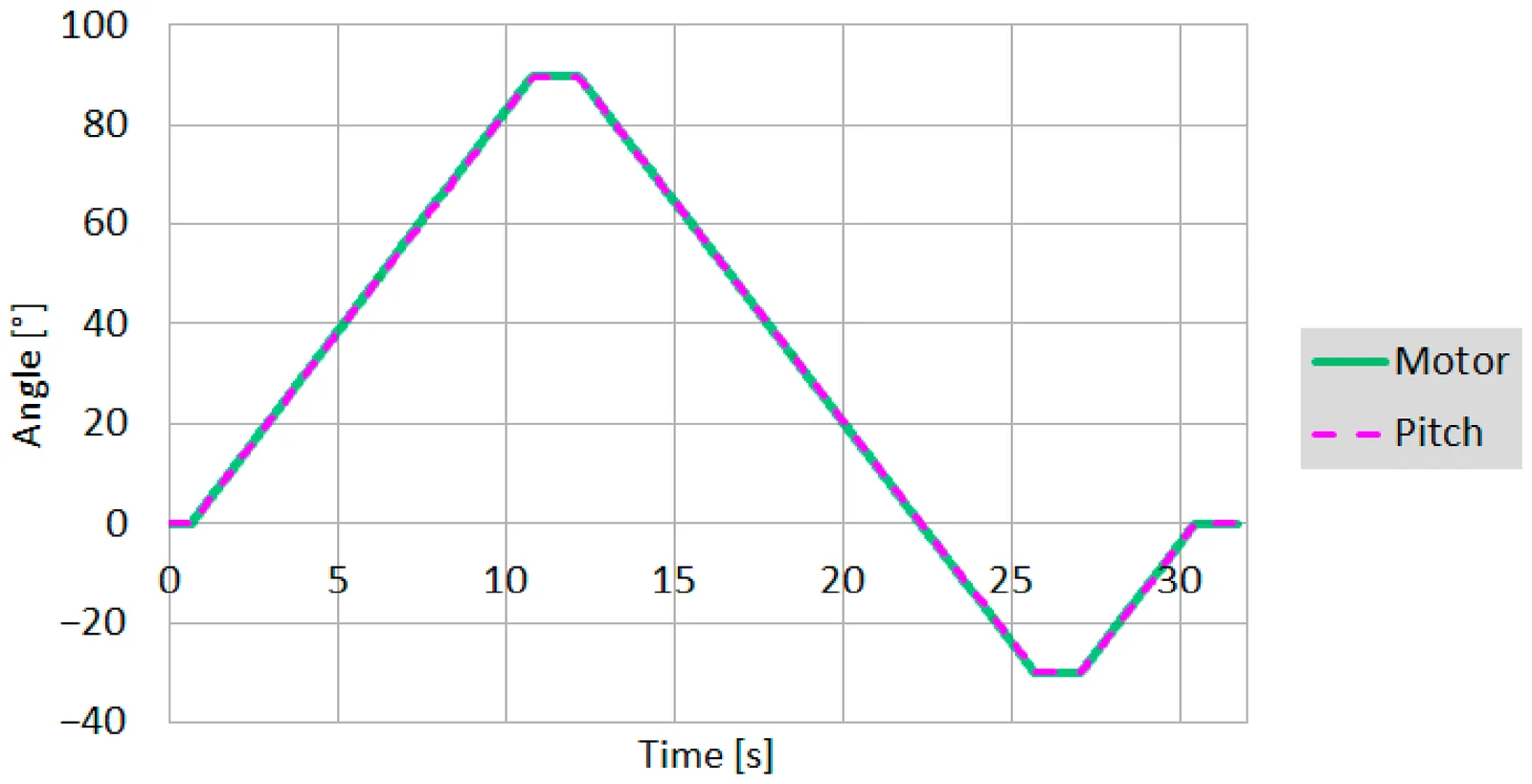
Measured angle compared to the reference trajectory under 20° lateral deviation.

**Figure 10:**
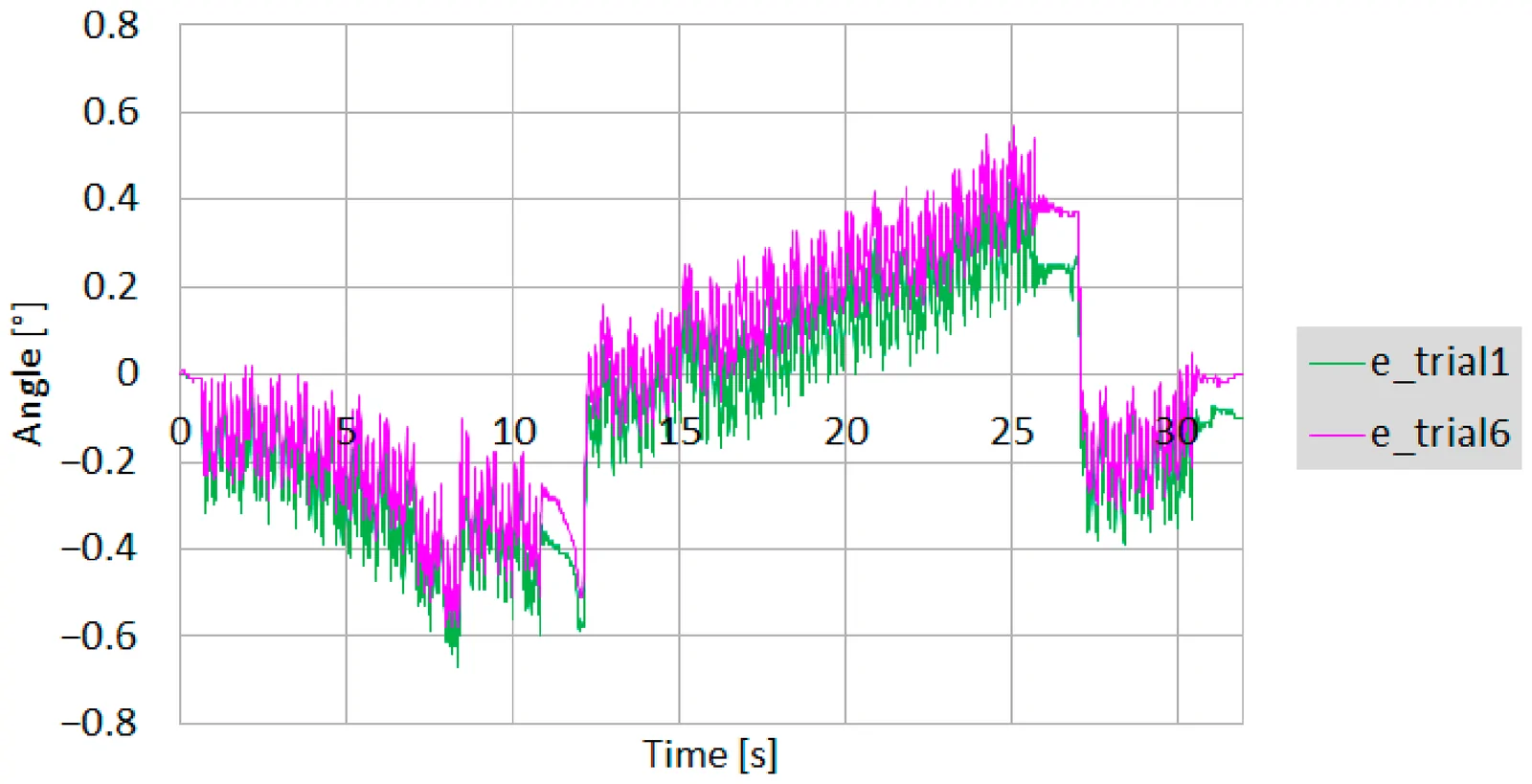
Measurement error *e_i_* profile during the 30 s movement cycle under 20° lateral deviation.

Increasing the initial lateral deviation to 20° tests whether cross-axis tilt leads to non-linear error accumulation. The measured pitch angle relative to the reference is shown in Figure 9, and the resulting errors *e_i_* are detailed in Figure 10. Increasing the lateral deviation to 20° did not cause proportional error growth: the mean MAE (0.221°) was close to the baseline value (0.214°). The largest values among the 10 trials were *e*_max_ = 0.670°, MAE = 0.237° and ME = −0.097°. The error showed the same pattern as in the previous cases: it increased gradually within each motion segment, persisted during the hold periods at +90° and −30° and changed sign at each direction reversal, without accumulating over the motion cycle.

### 3.4. Cross-Axis Angular Deviation of 30°

**Figure 11:**
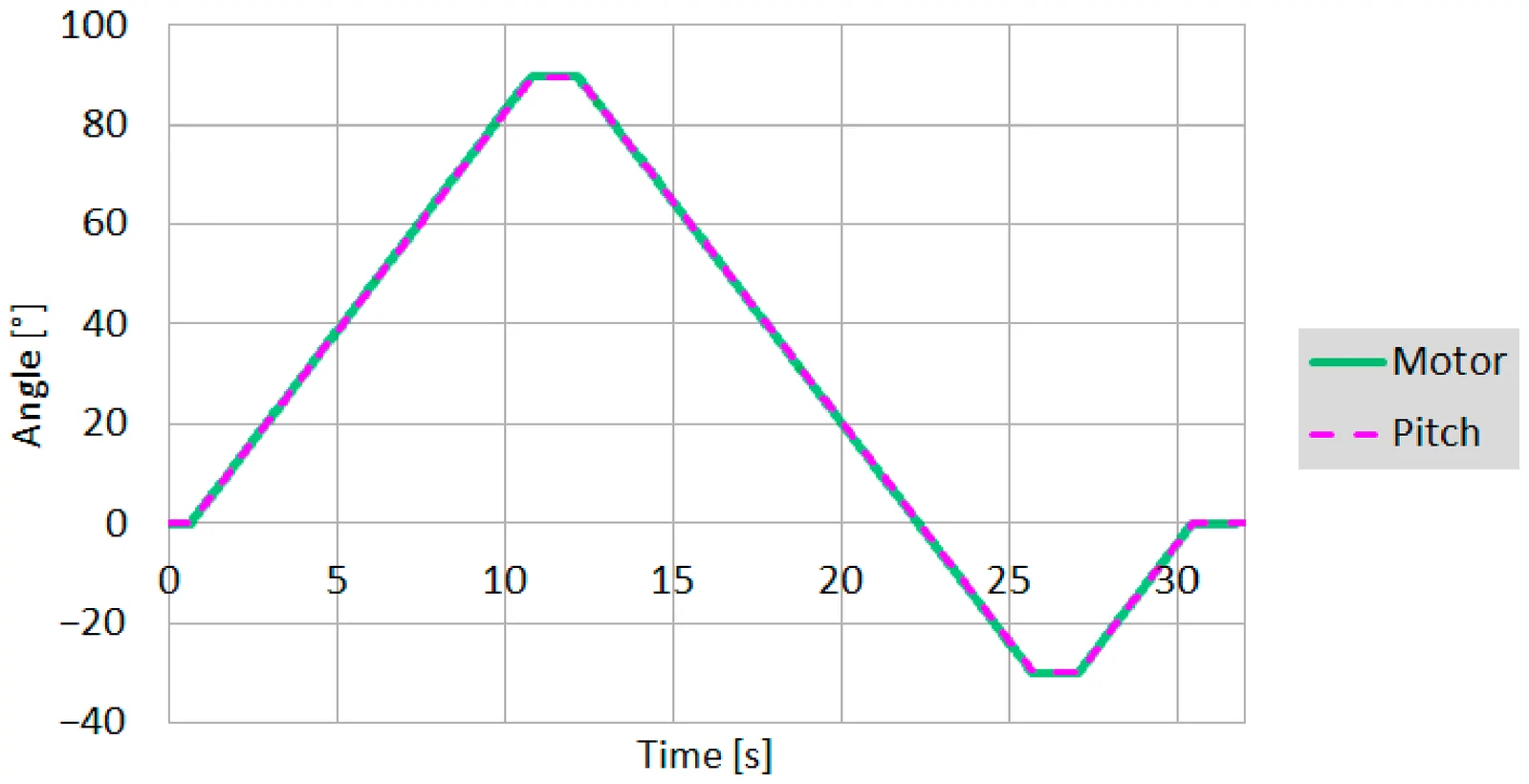
Measured angle compared to the reference trajectory under 30° lateral deviation.

**Figure 12:**
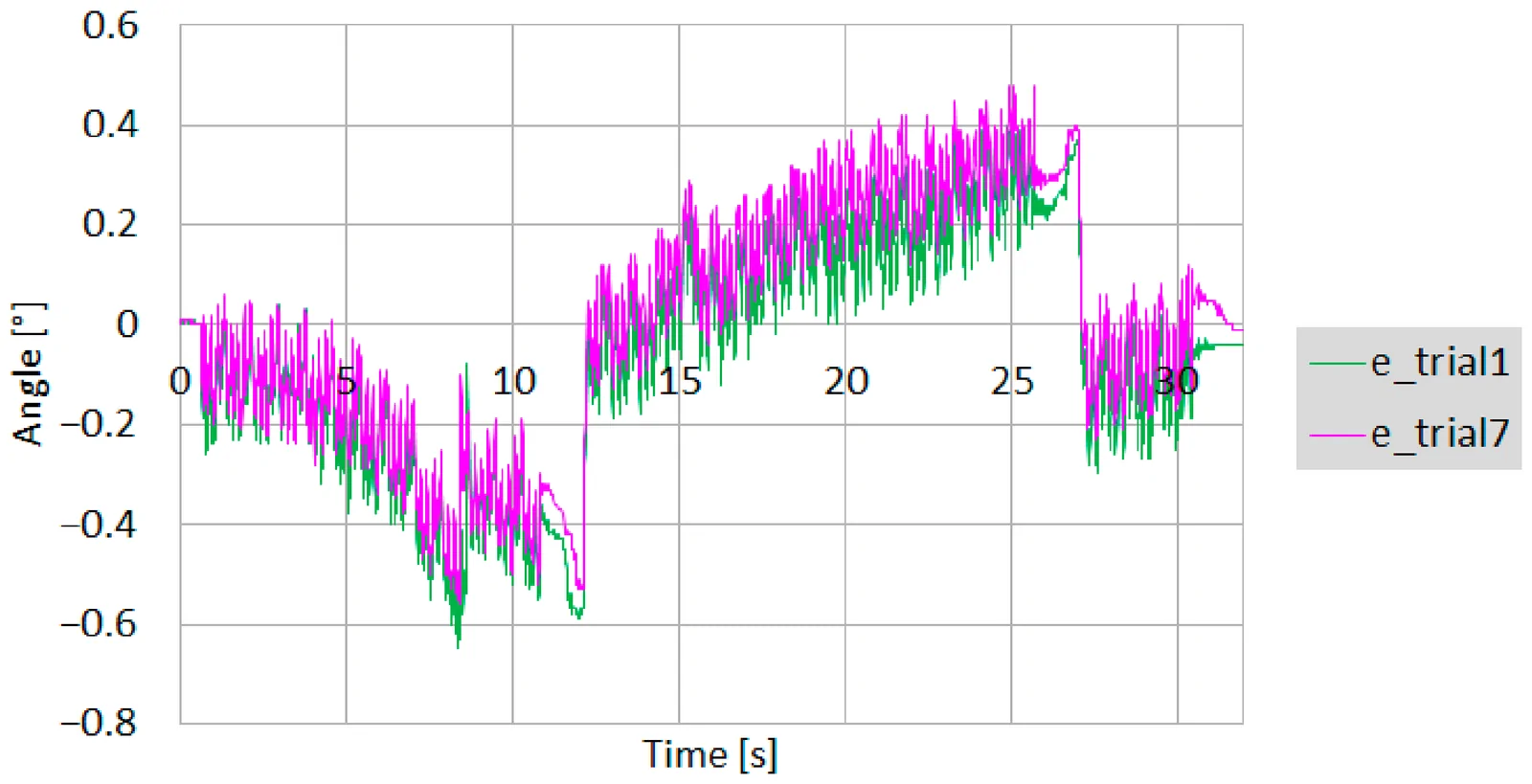
Measurement error *e_i_* profile during the 30 s movement cycle under 30° lateral deviation.

To determine operational boundaries under definite mounting offsets, testing was conducted at an initial lateral tilt of 30°. Higher offsets were not evaluated because lateral roll angles exceeding 30° generally would surpass safe operating limits for mobile machinery and represent imminent tip-over conditions in field environments. Pitch angle tracking is presented in Figure 11, while the errors are shown in Figure 12. Consistent with the previous conditions, no degradation of the pitch-tracking performance was observed under the 30° lateral deviation, and the error profile (Figure 12) showed the same pattern as in the earlier runs. The largest values among the 10 trials were *e*_max_ = 0.650°, MAE = 0.228° and ME = −0.065°. The MAE remained below 0.23° in all trials and the peak errors below 1.0°, and the error did not accumulate over the motion cycle.

### 3.5. Preliminary Mobile Robot Field Validation

**Figure 13:**
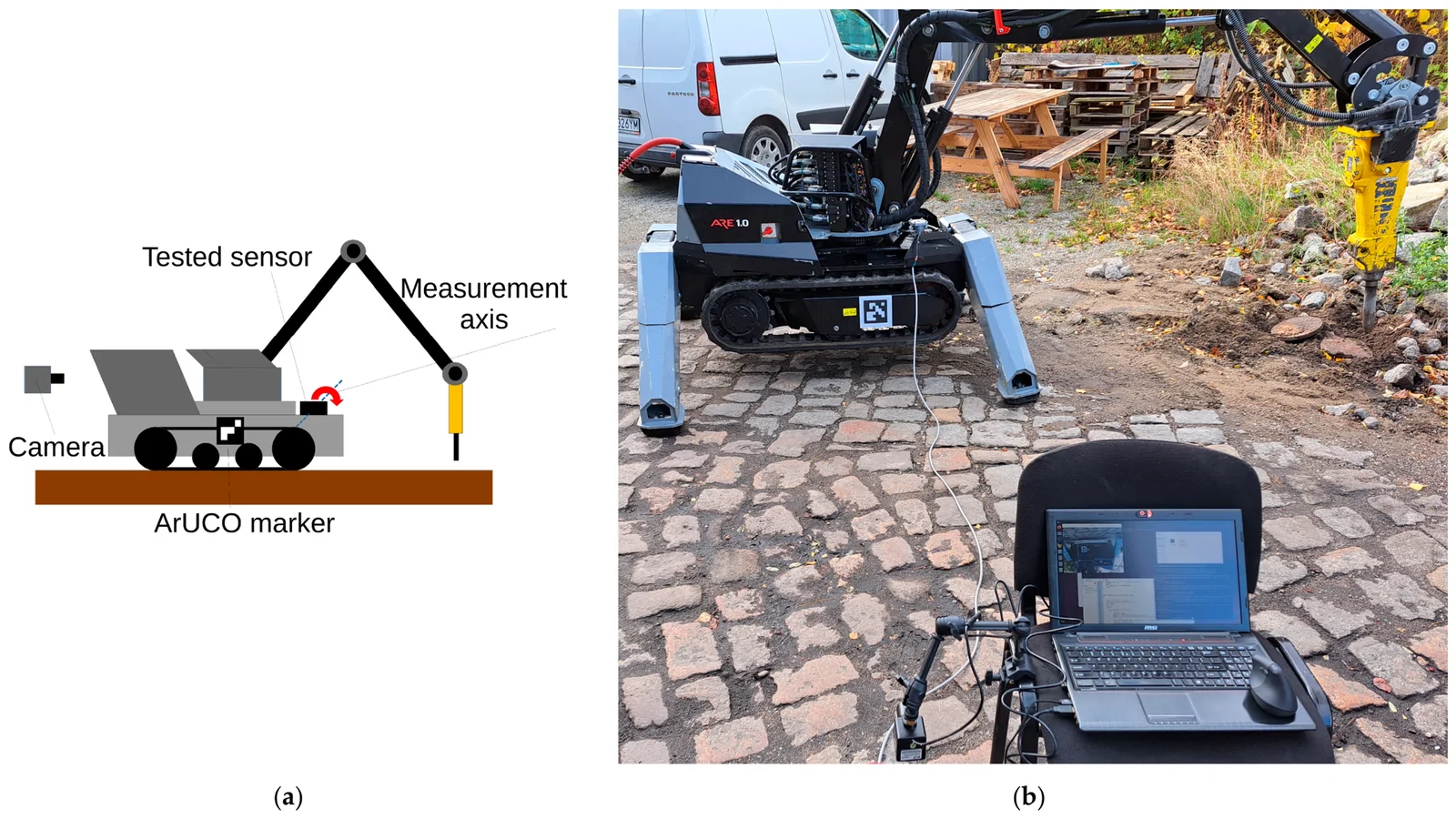
Mobile demolition robot field test: (**a**) Setup scheme. (**b**) Physical platform equipped with the JD1121 inclinometer and ArUco code for vision reference.

To assess the sensor performance in field applications, preliminary tests were executed on a mobile demolition robot (ARE 1.0, Advanced Robotic Engineering Sp. z o.o., Wrocław, Poland) changing chassis pitch orientation by pressing its manipulator with inactive tool against the ground. The experimental setup, featuring the chassis-mounted JD1121 inclinometer and an ArUco marker, which was tracked by an external camera to determine reference pitch angle data, is shown in Figure 13a, while the physical prototype used for the experiment is presented in Figure 13b.

**Figure 14:**
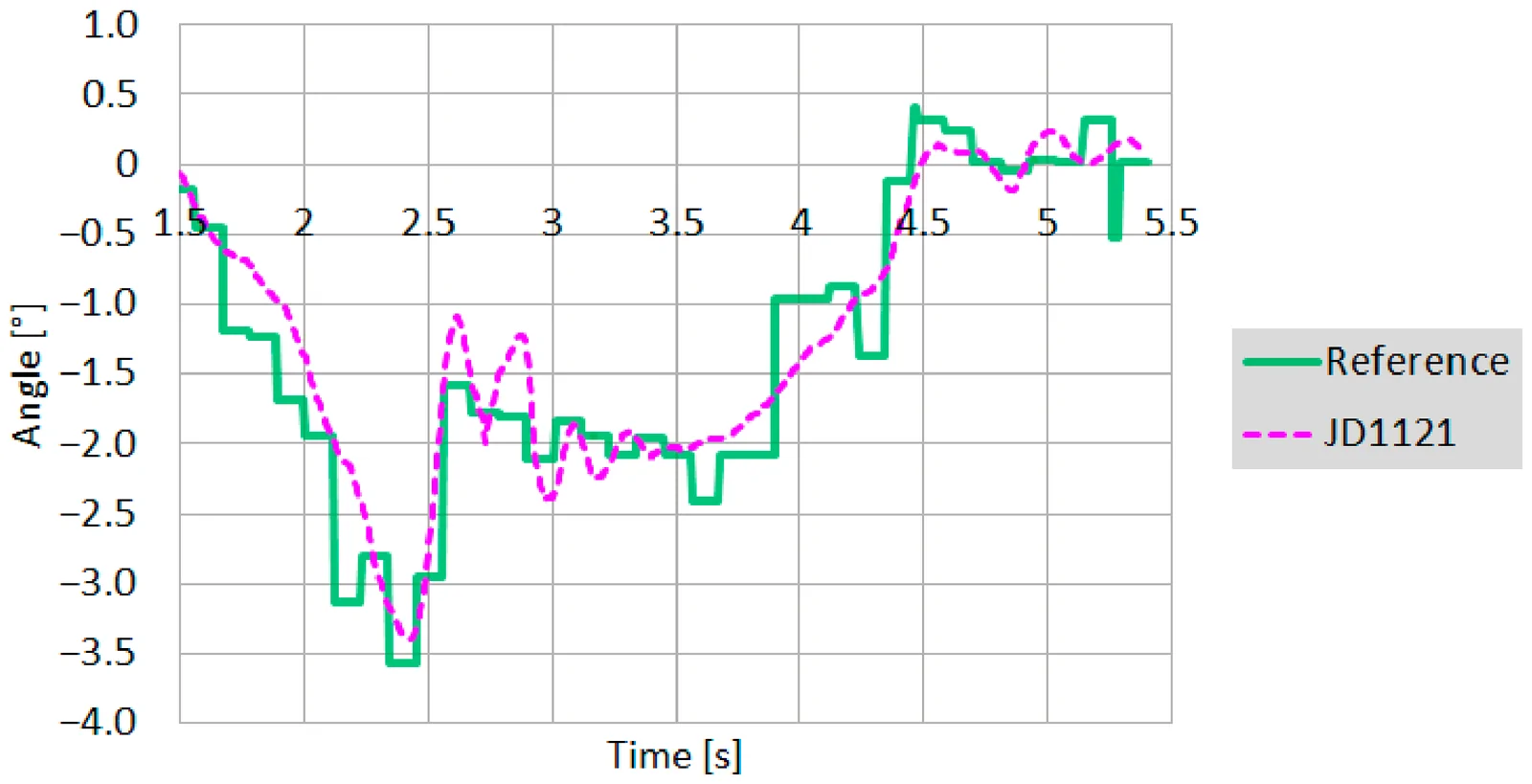
Demolition robot field test-angle comparison between the JD1121 inclinometer output and the ArUco optical reference.

**Figure 15:**
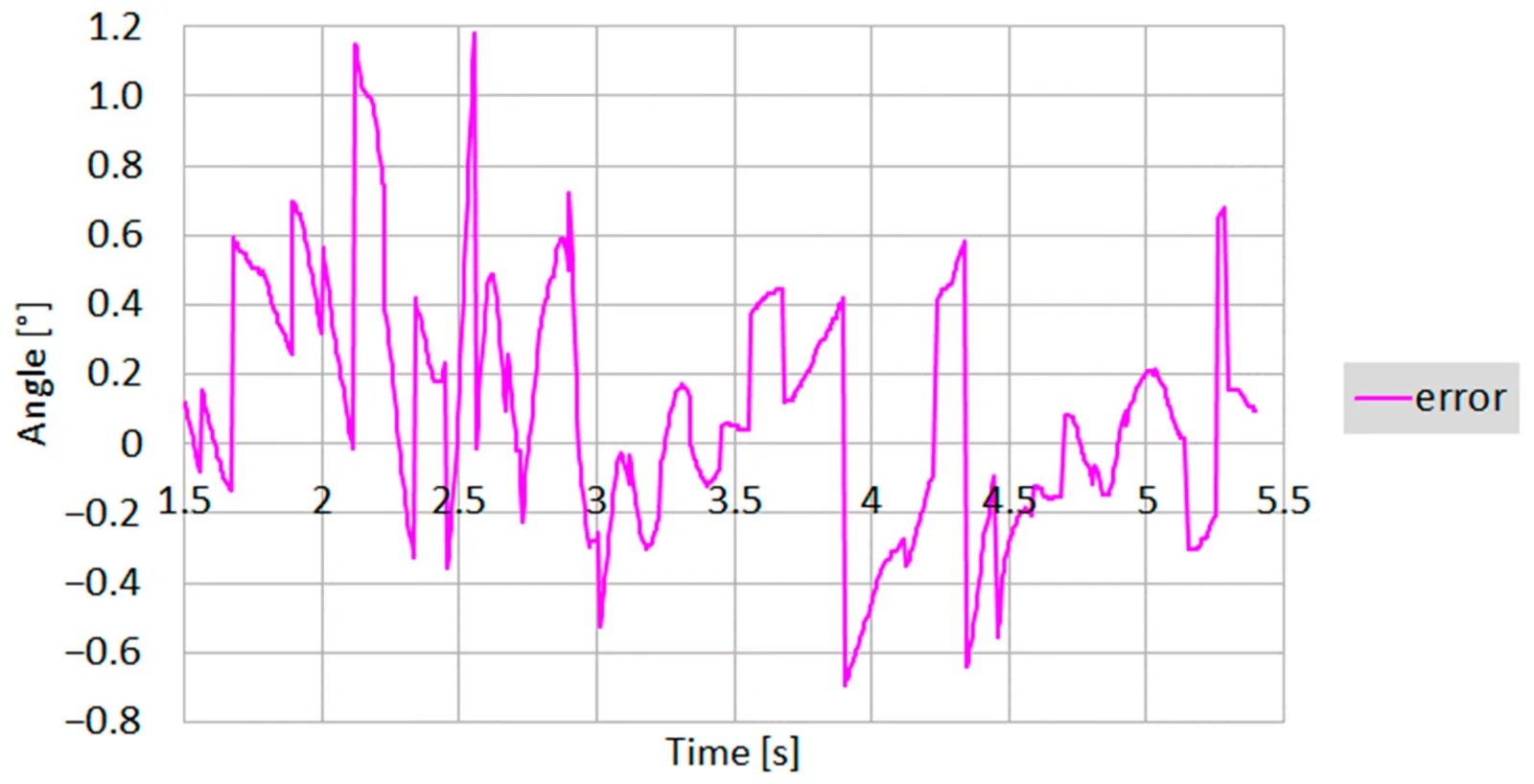
Measurement error *e_i_* recorded during mobile demolition robot field validation.

The continuous pitch tracking profile recorded during manipulator-induced angular movement is presented in Figure 14, with the corresponding instantaneous error *e_i_* shown in Figure 15. No filtering was applied to the data. The analysis was limited to the motion phase of the recording: its start and end were identified manually as the first and last instants at which the optical reference angle exceeded the range of fluctuations observed at rest, and the rest phases before and after the motion were excluded. During the evaluated trial, the JD1121 inclinometer closely tracked the optical reference trajectory, with minor delays and overshoots visible in Figure 14, yielding MAE = 0.287° and ME = 0.097°. Unlike the laboratory runs with pre-planned motor trajectories, the field test was manually controlled by an operator, resulting in an irregular motion profile. In addition, the ArUco optical reference exhibits a step-like discrete pattern (Figure 14), probably related to the camera sampling rate and resolution, which may contribute to short-term error spikes (Figure 15). The maximum error reached *e*_max_ = 1.180°, slightly exceeding the laboratory values. These results provide a preliminary validation of on-platform orientation tracking under real operating conditions; they do not constitute a validation of a complete anti-tip function, which will require comprehensive field testing, including heavy-duty demolition operations with high-frequency vibrations.

## 4. Discussion

The experimental evaluation conducted in this study provides a comprehensive performance assessment of the IFM JD1121 single-axis industrial inclinometer under dynamic pitch motion and static cross-axis angular deviations. By combining controlled multi-trial laboratory testing on a mechatronic rig with preliminary field testing on a demolition robot, the analysis addresses both metrological accuracy and practical implementation for mobile machine stability monitoring.

The results of all 40 laboratory trials show consistent measurements and a limited influence of the lateral deviation on the pitch-tracking performance. In all conditions, the inclinometer output closely followed the reference profile, and the error showed the same pattern: a gradual increase within each motion segment and an abrupt change in sign at each direction reversal. The individual numerical results, as well as the averaged statistical metrics (means) across the 10 automated trials for each experimental case are presented in Table 2. Examining the four planned distinct experimental conditions reveals that in the flat surface (0°) baseline test, the peak errors reached *e*_max_ = 1.060°, and MAE = 0.335°. Introducing a static initial 10° roll tilt yielded a maximum trial error of *e*_max_ = 0.760° with localized static offsets reaching about 0.5° near −30° pitch. At a 20° offset, accuracy remained stable with *e*_max_ = 0.670° and MAE = 0.237°, while the 30° roll tilt condition maintained limited errors with *e*_max_ = 0.650° and MAE = 0.228°.

**Table 2:** Numerical results and statistical metrics averaged across 10 automated trials per each setup case of cross-axis angle deviation (means, SD, 95% confidence interval of the mean, *n* = 10).

No.	Deviation 0° Case			Deviation 10° Case			Deviation 20° Case			Deviation 30° Case		
	*e*_max_ [°]	MAE [°]	ME [°]	*e*_max_ [°]	MAE [°]	ME [°]	*e*_max_ [°]	MAE [°]	ME [°]	*e*_max_ [°]	MAE [°]	ME [°]
1.	1.060	0.335	−0.148	0.760	0.299	−0.144	0.670	0.220	−0.097	0.650	0.202	−0.065
2.	0.590	0.199	−0.010	0.640	0.239	0.001	0.560	0.212	−0.018	0.610	0.207	−0.014
3.	0.600	0.200	0.024	0.650	0.268	0.021	0.620	0.237	−0.033	0.590	0.214	0.001
4.	0.630	0.212	0.011	0.730	0.276	0.059	0.580	0.218	0.004	0.570	0.203	0.019
5.	0.620	0.192	0.027	0.680	0.264	−0.001	0.590	0.220	0.013	0.570	0.210	0.007
6.	0.620	0.204	0.010	0.680	0.261	0.004	0.580	0.221	−0.019	0.590	0.206	−0.016
7.	0.640	0.216	0.005	0.720	0.258	0.005	0.580	0.220	−0.018	0.560	0.204	−0.016
8.	0.580	0.188	0.008	0.710	0.256	0.016	0.660	0.227	0.020	0.540	0.195	0.013
9.	0.630	0.186	−0.002	0.670	0.242	−0.004	0.630	0.218	0.024	0.600	0.210	−0.016
10.	0.640	0.207	−0.006	0.700	0.271	0.020	0.640	0.217	−0.009	0.600	0.228	0.004
Mean	0.661	0.214	−0.008	0.694	0.263	−0.002	0.611	0.221	−0.013	0.588	0.208	−0.008
SD	0.142	0.044	0.050	0.037	0.017	0.053	0.038	0.007	0.035	0.030	0.009	0.024
95% CI low	0.560	0.183	−0.044	0.667	0.251	−0.040	0.584	0.216	−0.038	0.566	0.202	−0.025
95% CI high	0.762	0.245	0.028	0.721	0.275	0.036	0.638	0.226	0.012	0.610	0.214	0.009

The mean ME remained negligible across all test series (−0.013° to −0.002°), indicating that the lateral inclination introduced no measurable directional bias. The mean MAE ranged from 0.208° to 0.263°. The mean *e*_max_ was 0.661° at 0°, 0.694° at 10°, 0.611° at 20° and 0.588° at 30°; it differed between the conditions (Table 2), but showed no increasing trend with lateral deviation, and none of the pairwise differences from 0° was significant (Table 3). Across the entire laboratory dataset, the largest error was *e*_max_ = 1.060° (trial 1 at 0°), while all mean *e*_max_ values remained below 0.7°. The cross-axis sensitivity coefficient was *S*_CA_ = −0.0001 °/° (95% CI [−0.0012, 0.0010] °/°, *p* = 0.84), i.e., |*S*_CA_| ≤ 0.12%, corresponding to a change in the mean error below 0.04° over the tested range of 0–30°.

The measured MAE values should be interpreted in the light of two considerations. First, the manufacturer’s static accuracy of ±0.3° was used only as an engineering reference and as the equivalence margin for assessing whether the lateral deviation changed the sensor performance to a practically relevant extent; as it refers to static conditions, the dynamic tests do not directly validate it. For dynamic conditions, the manufacturer specifies an accuracy of ±0.5°. Second, the expanded uncertainty of the angular reference (*U* = 0.17°, Table 1) is of the same order as the measured MAE values. The reported errors therefore include a contribution of the reference and should be regarded as conservative (upper) estimates of the inclinometer error; assuming independent, approximately normally distributed error sources, this contribution does not exceed approximately 0.02°. Since the same test stand and motion profile were used in all conditions, the reference error is largely common to them and partly cancels in the comparison between conditions; this assumes that the lateral deviation did not change the behavior of the drive, as the reference was validated at 0° lateral deviation.

In order to further statistically evaluate the results, means ± SD (standard deviation) with 95% confidence intervals are reported in Table 2. Differences among the conditions were tested with Welch’s ANOVA, confirmed by the Kruskal–Wallis test, and each condition was compared with 0° using Welch’s *t*-test with Holm correction (effect size: Hedges’ g) (Table 3). Practical equivalence with 0° was assessed by two one-sided tests (TOST) with a margin of ±0.3°, i.e., the static accuracy specified by the manufacturer; a stricter margin of ±0.05° (specified repeatability) was evaluated additionally. The significance level was *α* = 0.05.

Lateral deviation had an effect on MAE (Welch *F*(3, 18.4) = 26.1, *p* < 0.001) and *e*_max_ (*F*(3, 19.3) = 15.9, *p* < 0.001), but not on ME (*F*(3, 19.0) = 0.10, *p* = 0.96); the Kruskal–Wallis test led to the same results. Compared with 0°, MAE increased by 0.049° at 10° (95% CI [0.017, 0.082]°, *p*_Holm_ = 0.018, *g* = 1.43), while the differences at 20° and 30° were not significant (Table 3 and Table 4). The effect was thus not monotonic with respect to the lateral angle. None of the pairwise differences in *e*_max_ was significant. All conditions were equivalent to 0° within ±0.3° for all metrics (TOST, *p*_Holm_ < 0.001); within ±0.05°, equivalence was confirmed for ME in all conditions and for MAE at 20° and 30°, but not for MAE at 10° (90% CI of the difference [0.023, 0.076]°) nor for *e*_max_. Thus, within the ±0.3° equivalence margin derived from the manufacturer’s static accuracy, the performance under lateral deviation was practically equivalent to the 0° baseline.

To assess possible time-dependent effects within the series, the trend of the error metrics over trials 2–10 was evaluated with the Spearman rank correlation. No monotonic trend of MAE or ME was found in any series (*p* ≥ 0.16); for *e*_max_, a positive rank correlation was found at 20° (*ρ* = 0.70, *p* = 0.04), which was not significant after Holm correction across the four series. Excluding the first trial of each series yielded mean MAE values of 0.200°, 0.259°, 0.221° and 0.209° for 0°, 10°, 20° and 30°, respectively, and did not change the results of the equivalence tests within ±0.3°.

These results quantify the influence of lateral deviation on the pitch-tracking performance of the inclinometer: the lateral deviation affected MAE, but did not introduce a persistent pitch-angle bias. This is consistent with reports that multi-axis angular motion can influence MEMS inclination readings [25].

In all conditions, including 0°, the error showed the same pattern: it increased gradually within each motion segment, reaching its largest values near the extreme pitch amplitudes (+90° and −30°), persisted during the stationary hold phases and changed sign abruptly at each direction reversal; the mean *e*_max_ showed no increasing trend with lateral deviation. As this pattern followed the direction of the preceding motion and was present already at 0°, it is not attributable to the lateral deviation; its origin was not investigated in this study. The variation in the dwell deviation at −30° observed in the reference validation may have contributed to the offsets during the hold phases. The first trial of each configuration exhibited a larger negative ME. Its cause could not be identified unambiguously, though possible contributors include start-up effects of the test stand. Trials 2–10 showed consistent performance with a small systematic bias.

In contrast to the conventional 3-axis IMU evaluated in our earlier work [33], the single-axis inclinometer provides a simpler architecture free from integration drift, magnetometer susceptibility to surrounding metallic structures, or gimbal lock singularities. These characteristics are relevant when transitioning from laboratory to real-world application settings, such as the preliminary field test on the mobile demolition robot presented here. In this test, with an operator-controlled, irregular motion profile, the maximum error reached *e*_max_ = 1.180°, slightly above the largest value observed in the laboratory tests (1.060°), while MAE = 0.287° and ME = 0.097°. Whether this accuracy is sufficient for real-time anti-tip safety monitoring depends on the stability margin required by the specific application and remains to be verified in future field tests.

## 5. Conclusions

This study evaluated the pitch-angle tracking performance and cross-axis resilience of the IFM JD1121 single-axis industrial inclinometer, with a view to its future use in stability monitoring of mobile machinery. The sensor was tested on a stepper-motor-driven test rig with an optically validated angular reference (Section 2) under fixed lateral deviations of 0°, 10°, 20° and 30° (10 trials each), and in a preliminary field test on a mobile demolition robot.

Within the tested range, lateral deviation did not degrade the tracking performance beyond the specified accuracy of the sensor. The mean MAE ranged from 0.208° to 0.263° and the mean error from −0.013° to −0.002°, with no effect of lateral deviation on ME. The largest increase in MAE (0.05° at 10°) was statistically significant but non-monotonic with respect to the lateral angle. Furthermore, this increase was about one order of magnitude smaller than the manufacturer’s static accuracy of ±0.3°, which served here as an engineering reference and equivalence margin rather than as a quantity directly validated by the dynamic tests. Owing to the fixed test sequence, this increase cannot be unambiguously attributed to the lateral deviation itself. Maximum errors reached 1.060°; in all conditions, the error increased within each motion segment and changed sign at direction reversals, a pattern already present at 0° and therefore not attributable to the lateral deviation. Since the expanded uncertainty of the angular reference (0.17°) is of the same order as the measured MAE, the reported errors should be regarded as conservative estimates. As the initial reading was subtracted in each trial, the results characterise relative angular tracking rather than absolute inclination accuracy.

In the preliminary field test, the maximum error with respect to the optical reference was 1.180° during manipulator-induced pitch changes. This test should be regarded as a preliminary validation of on-platform orientation tracking rather than of a complete anti-tip function. From a practical standpoint, the results indicate that the sensor is a viable inclination input candidate for tip-over stability monitoring of mobile machinery. Such use will additionally require calibration of the absolute zero offset and of the sensor mounting on the platform, as well as validation under real operating conditions.

Several limitations should be noted. A single sensor unit was tested, so unit-to-unit variability was not quantified. Only positive lateral deviations (0° to +30°) were tested, assuming lateral symmetry of the sensor, and the static offset induced by the lateral deviation at the initial position was not evaluated. The laboratory motion profile used a single nominal angular velocity of approximately 9°/s, representative of slow chassis pitch changes, so the behavior under fast rotations was not examined. The angular reference was validated at 0° lateral deviation only. In the reference validation, the deviation at the −30° dwell decreased over the first consecutive runs (from 0.19° to 0.03°); as the main measurement series were started without a preceding run-in of the test stand, a similar effect may have contributed to the larger errors in the first trial of each series. The conditions were tested in a fixed, non-randomised sequence. While the drift analysis showed no monotonic trend of MAE or ME within trials 2–10 of any series (*p* ≥ 0.16), it cannot exclude other time- or order-dependent effects, which therefore cannot be fully separated from the effect of lateral deviation. Finally, the field test was performed without operating the crushing tools or the hydraulic hammer, i.e., without severe high-frequency vibrations.

Future research will focus on expanded field testing across diverse driving manoeuvres, on randomised test sequences with a run-in phase of the test stand, and on evaluating the sensor performance under the intense high-frequency vibrations generated during hammering and concrete crushing, in both manual and autonomous operation.

## Figures and Tables

**Table 1:** Uncertainty budget of the angular reference (commanded stepper-motor position), based on the optical validation.

Component	Distribution	*u* [°]	Source
Step resolution (0.05625°)	rectangular	0.016	1.8°/32 microstep, quantisation ±½ step
Max. systematic dwell deviation	rectangular	0.055	largest run-averaged dwell deviation (0.095°, at +90°)
Dwell repeatability	normal	0.059	pooled SD of the −30° and +90° dwell deviations across runs
Zero return after full profile	normal	0.010	RMS deviation at 0° after the full profile
Direction-dependent hysteresis	rectangular	0.003	upper bound 0.005° from least-squares fit
Combined standard uncertainty *u*_ref_		0.08	
Expanded uncertainty *U* (*k* = 2)		0.17	

**Table 3:** Differences in the error metrics relative to the 0° condition (Δ = condition−0°). Welch’s *t*-test with Holm correction; equivalence assessed by TOST with margins of ±0.3° and ±0.05°.

Metric	Comparison	Δ [°]	95% CI of Δ [°]	*p*_Holm_	Hedges’ *g*	TOST ±0.3° *p*_Holm_	TOST ±0.05° *p*_Holm_
MAE	10° vs. 0°	+0.049	[0.017, 0.082]	0.018	1.430	<0.001	0.485
MAE	20° vs. 0°	+0.007	[−0.024, 0.039]	1.000	0.220	<0.001	0.017
MAE	30° vs. 0°	−0.006	[−0.037, 0.026]	1.000	−0.180	<0.001	0.017
ME	10° vs. 0°	+0.006	[−0.043, 0.054]	1.000	0.100	<0.001	0.035
ME	20° vs. 0°	−0.005	[−0.046, 0.036]	1.000	−0.120	<0.001	0.035
ME	30° vs. 0°	0.000	[−0.038, 0.038]	1.000	−0.010	<0.001	0.022
*e*_max_	10° vs. 0°	+0.033	[−0.070, 0.136]	0.612	0.310	<0.001	1.000
*e*_max_	20° vs. 0°	−0.050	[−0.153, 0.053]	0.612	−0.460	<0.001	1.000
*e*_max_	30° vs. 0°	−0.073	[−0.175, 0.029]	0.429	−0.680	<0.001	1.000

**Table 4:** Comparison of the four lateral-deviation conditions (0°, 10°, 20°, 30°; n = 10 trials each). Welch’s ANOVA with effect size ω^2^; Kruskal–Wallis test as a non-parametric check.

Metric	Welch *F*	df_1_	df_2_	*p*	*ω* ^2^	Kruskal–Wallis *H*(3)	*p*
MAE	26.100	3	18.400	<0.001	0.430	25.500	<0.001
ME	0.100	3	19.000	0.960	0	3.100	0.380
*e*_max_	15.900	3	19.300	<0.001	0.180	20.400	<0.001

## Data Availability

The data were provided by Advanced Robotic Engineering Sp. z o.o. and are available from the corresponding author with the permission of the company.

## Abbreviations

The following abbreviations are used in this manuscript:

ArUco	Augmented Reality University of Cordoba, marker for visual tracking
IMU	Inertial Measurement Unit
MAE	Mean Absolute Error
ME	Mean Error
PET-G	Polyethylene Terephthalate Glycol, material for 3D printing
ANOVA	Analysis of Variance
CAN	Controller Area Network
CI	Confidence Interval
MEMS	Micro-Electro-Mechanical Systems
SD	Standard Deviation
TOST	Two One-Sided Tests
ZMP	Zero-Moment Point

## References

[1] Jeng S.L., Yang C.F., Chieng W.H. (2010). Outrigger force measure for mobile crane safety based on linear programming optimization. Mech. Based Des. Struct. Mach..

[2] Jin S., Kang L., Peng Y.-C., He Z., Song R., Qian L., Zhang L. Autonomous excavator system for construction earth moving. Proceedings of the 2nd Future of Construction Workshop at the International Conference on Robotics and Automation (ICRA).

[3] Lee H.J., Brell-Cokcan S. (2023). Towards controlled semi-autonomous deconstruction. Constr. Robot..

[4] Park H., Langari R., Yi H. (2024). Design and testing of double-wishbone suspension for enhanced outdoor maneuver stability of a six-wheeled mobile robot. Mechatronics.

[5] Kosiara A. (2014). Methodology of Identification of Single Bucket Excavator’s Selected Parameters for Its Microprocessor System of Stability Control. Logistyka.

[6] Wiehagen W.J., Mayton A.G., Jaspal J.S., Turin F.C. (2001). An Analysis of Serious Injuries to Dozer Operators in the U.S. Mining Industry.

[7] Garcia E., Arevalo J.C., de Santos G., Gonzalez de Santos P. (2002). A comparative study of stability margins for walking machines. Robotica.

[8] Roan P.R., Burmeister A., Rahimi A., Holz K., Hooper D. Real-world validation of three tipover algorithms for mobile robots. Proceedings of the 2010 IEEE International Conference on Robotics and Automation (ICRA).

[9] Wu J., Guzzomi A.L., Hodkiewicz M.R. (2014). Static stability analysis of non-slewing articulated mobile cranes. Aust. J. Mech. Eng..

[10] Papadopoulos E.G., Rey D.A. (2000). The force-angle measure of tipover stability margin for mobile manipulators. Veh. Syst. Dyn..

[11] Li Y., Liu Y. A new task-consistent overturn prevention algorithm for redundant mobile modular manipulators. Proceedings of the 2005 IEEE/RSJ International Conference on Intelligent Robots and Systems (IROS).

[12] Duda J. (2017). A Lyapunov functional for a system with both lumped and distributed delay. Arch. Control Sci..

[13] Vaidyanathan S., Sambas A., Mamat M., Sanjaya W.S.M. (2017). A new three-dimensional chaotic system with a hidden attractor, circuit design and application in wireless mobile robot. Arch. Control Sci..

[14] Di Massa G., Malfi P., Pagano S., Rocca E., Savino S. (2026). Analysis, Modeling, and Simulation of a Rocker-Bogie System Overcoming a Harmonic Bump. Machines.

[15] Ryu S., Won J., Chae H., Kim H.S., Seo T. (2024). Evaluation Criterion of Wheeled Mobile Robotic Platforms on Grounds: A Survey. Int. J. Precis. Eng. Manuf..

[16] Tao Y., Liu H., Song Y., Deng C., Wang B., Zheng P. (2025). Dynamic Tip-Over Avoidance Method for Mobile Manipulators Based on the Extended Zero-Moment Point Algorithm for Human-Robot Collaboration. J. Comput. Inf. Sci. Eng..

[17] Ding X., Liu Y., Hou J., Ma Q. (2019). Online Dynamic Tip-Over Avoidance for a Wheeled Mobile Manipulator with an Improved Tip-Over Moment Stability Criterion. IEEE Access.

[18] Kim T., Lee D., Kwon G., Yang H. (2025). Terrain-Adaptive Mobile-Base Platform for Enhanced Robot Adaptability and Stability. IEEE Access.

[19] Tian H.-B., Wang A., Lu M., Zhang M., Zhang Z., Zhang W., Su X. (2024). Stability Analysis and Control Method of a Variable Structure Detection Robot for Underground Rescue. Mech. Sci..

[20] Fiedeń M. (2025). Kinematic analysis of an omnidirectional tracked vehicle. Appl. Sci..

[21] Zhu Q., Xiao C., Hu H., Liu Y., Wu J. (2018). Multi-Sensor Based Online Attitude Estimation and Stability Measurement of Articulated Heavy Vehicles. Sensors.

[22] Samatas G.G., Pachidis T.P. (2022). Inertial Measurement Units (IMUs) in mobile robots over the last five years: A review. Designs.

[23] Ursel T., Olinski M. (2021). Displacement estimation based on optical and inertial sensor fusion. Sensors.

[24] Ahmad N., Ghazilla R.A.R., Khairi N.M., Kasi V. (2013). Reviews on various inertial measurement unit (IMU) sensor applications. Int. J. Signal Process. Syst..

[25] Yang Q., Cai C., Yang M., Kong M., Liu Z., Liang F. (2023). Dynamic tilt testing of MEMS inclinometers based on conical motions. Metrol. Meas. Syst..

[26] Komarizadehasl S., Komary M., Alahmad A., Lozano-Galant J.A., Ramos G., Turmo J. (2022). A Novel Wireless Low-Cost Inclinometer Made from Combining the Measurements of Multiple MEMS Gyroscopes and Accelerometers. Sensors.

[27] Hoang M.L., Pietrosanto A. (2021). A new technique on vibration optimization of industrial inclinometer for MEMS accelerometer without sensor fusion. IEEE Access.

[28] Hoang M.L., Pietrosanto A. (2021). A robust orientation system for inclinometer with full-redundancy in heavy industry. IEEE Sens. J..

[29] Dube C. Experimental validation of tip over stability of a tracked mobile manipulator. Proceedings of the 2013 IEEE AFRICON.

[30] Liu Z., Kim J.I. (2025). Autonomous Earthwork Machinery for Urban Construction: A Review of Integrated Control, Fleet Coordination, and Safety Assurance. Buildings.

[31] Ramalingam N., Yang H., Lim K.S., Ahmad H. (2024). Cantilever-based biaxial FBG inclinometer with low cross-axis sensitivity. Opt. Appl..

[32] Li X.-A., Zhang D., Ning G., Wang F., Jia X. (2027). Physical modeling and reliability analysis of industrial robot motion smoothness considering dataset variability. Reliab. Eng. Syst. Saf..

[33] Fiedeń M., Olinski M., Szrek J., Bałchanowski J., Ceccarelli M. (2026). Comparative performance of inertial sensors for mobile robot stability monitoring. New Trends in Mechanism and Machine Science (EuCoMeS 2026).

